# Assessing Representation Learning and Clustering Algorithms for Computer-Assisted Image Annotation—Simulating and Benchmarking MorphoCluster

**DOI:** 10.3390/s22072775

**Published:** 2022-04-04

**Authors:** Simon-Martin Schröder, Rainer Kiko

**Affiliations:** 1 Department of Computer Science, Kiel University, 24118 Kiel, Germany; 2 Laboratoire d’Océanographie de Villefranche, Sorbonne Université, 06230 Villefranche-sur-Mer, France; rainer.kiko@obs-vlfr.fr

**Keywords:** image annotation, machine learning, representation learning, clustering, biological oceanography

## Abstract

Image annotation is a time-consuming and costly task. Previously, we published MorphoCluster as a novel image annotation tool to address problems of conventional, classifier-based image annotation approaches: their limited efficiency, training set bias and lack of novelty detection. MorphoCluster uses clustering and similarity search to enable efficient, computer-assisted image annotation. In this work, we provide a deeper analysis of this approach. We simulate the actions of a MorphoCluster user to avoid extensive manual annotation runs. This simulation is used to test supervised, unsupervised and transfer representation learning approaches. Furthermore, shrunken *k*-means and partially labeled *k*-means, two new clustering algorithms that are tailored specifically for the MorphoCluster approach, are compared to the previously used HDBSCAN*. We find that labeled training data improve the image representations, that unsupervised learning beats transfer learning and that all three clustering algorithms are viable options, depending on whether completeness, efficiency or runtime is the priority. The simulation results support our earlier finding that MorphoCluster is very efficient and precise. Within the simulation, more than five objects per simulated click are being annotated with 95% precision.

## 1. Introduction

The annotation of images is a central step in many disciplines, including marine ecology [1], medicine [2,3], astronomy [4], face recognition [5] and machine learning [6]. Considerable progress has been made in the last years regarding the automation of image classification via machine learning approaches, especially since the breakthrough of convolutional neural networks (CNNs) [7]. One example where particularly many, diverse images are being acquired is the field of aquatic plankton research [8]. Specialized in situ cameras (such as the UVP5 [9]) or benchtop imaging systems (such as the ZooScan [10]) are being deployed to yield digital images of plankton (organisms drifting in the water column that can be caught with a slowly towed net) and particles. The commercialization and use of these tools by many users leads to a steadily growing influx of new image data. Classification of these images is needed, as the different photographed organisms and particles can perform very different functions in the marine ecosystem. Phytoplankton organisms are autotrophic and use sunlight, nutrients and carbon dioxide to build up organic biomass, whereas other organisms called zooplankton are heterotrophic and mostly feed on phytoplankton. The abundances of phyto- and zooplankton are shaped by complex physical, biological and chemical processes. The plankton distribution is therefore highly dynamic, “patchy” [11,12,13] and varies on large spatial and temporal scales [14,15,16]. As a result, the training sets required to train a classifier generally deviate from the distribution of newly observed samples (dataset shift or population drift) [17]. This can introduce a bias into the classification, if, e.g., a training set from the Mediterranean sea would be used to predict the classifications for an Arctic dataset. Carrying this out can distort the abundance estimates, in some cases, to a severe extent [18]. Therefore, machine classification is often followed by a manual validation of individual annotations to obtain high, human-level precision and a higher throughput. In the case of a high prevalence of novel classes or a strong dataset shift, the human workload is still considerable. Researchers, therefore, explored other ways to annotate marine data that are centered around efficiency [19,20,21].

In 2020, we proposed MorphoCluster [22] as another solution for this problem. We will refer to the first implementation and evaluation of MorphoCluster as MC20. MorphoCluster is a data-driven approach to accelerate the sorting of marine particles and plankton. It was designed especially for larger datasets with millions of objects, whereas it could be less effective for smaller datasets of only a few hundred objects. Several authors mention that MorphoCluster could be a promising approach to annotate large plankton datasets [23,24,25]. Efficiency in the MorphoCluster approach is maximized using two orthogonal techniques: first, similar objects are grouped into homogeneous clusters and the user validates the objects of each cluster jointly. Second, the annotation expert adds additional visually similar objects to the previously validated clusters.

**Figure 1:**
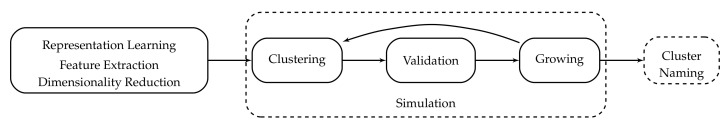
Sequence of operations in the MorphoCluster approach. The simulation is not part of the MorphoCluster application but allows us to examine different algorithmic configurations. In this work, we focus on the representation learning and the iterative annotation phase and omit the final naming step. We compare different approaches for representation learning, dimensionality reduction and clustering.

The general work flow of MorphoCluster comprises representation learning, feature extraction, dimensionality reduction, clustering, the validation and growing of clusters and cluster naming, as outlined in Figure 1.

In the initial representation learning [26] phase, a deep feature extractor is trained to project the complex morphology of an object depicted in an image into a metric vector space of limited dimensionality that facilitates the partitioning of the dataset into visually distinct and homogeneous clusters and allows the separation of different morphologies. Thereafter, the dimensionality of the image representations is reduced to minimize the computation time and storage requirements during the following, interactive steps.

During the subsequent iterative phase, similar objects are combined in homogeneous clusters. First, the image representations are clustered to extract groups of similar-looking objects. In the following cluster validation step, the annotation expert confirms visually pure clusters and rejects mixed clusters. In the subsequent cluster-growing step, the user expands the initially small clusters by determining their proper boundaries using an efficient exponential search. This similarity search is based on the Euclidean distances of the respective cluster centers and image representations. The objects that are not assigned to a cluster after the growing step are re-clustered, and the cluster approval and growth steps are repeated. Finally, the identified clusters are hierarchically arranged using agglomerative clustering to group similar clusters, which can be manually merged and named.

Overall, only a fraction of all images needs to be checked manually by the user, and the classification task is broken up into several more simple steps that only require the user to decide if a set of images is alike. Only in the final step is the more complex task to provide a classification for each object conducted; however, it is conducted on possibly thousands of images per decision.

In this work, we compare different implementations of the representation learning phase and the iterative clustering and annotation phase of the MorphoCluster approach. The final classification step will be examined and optimized in future work. To enable our analysis, we implemented a simulation framework that allows us to compare different algorithmic choices without the need to annotate whole datasets multiple times. As our analysis requires a large number of complete annotation trials and the single existing annotation in MC20 [22] already took more than 70 h, this would have been infeasible. Using this simulation framework, we compare several feature extraction techniques under different assumptions of label and data availability to enable a broader use of MorphoCluster also on new image types, for which, no prior annotations exist. Likewise, we test two novel semi-supervised clustering algorithms tailored specifically for the MorphoCluster approach. We give recommendations on the best configuration, depending on the availability of labeled training data and computational constraints.

## 2. Methods

### 2.1. Simulation of the MorphoCluster Process

To enable the comparison of different algorithmic choices, we simulated the operations of a MorphoCluster user in silico. For that, we used the expert annotations provided with the dataset as gold standard, which are otherwise hidden from other parts of the process. This provided us with an approximation of the behavior of a real user. The simulation acts on previously calculated image representations and consists of three components: the clusterer implements the clustering step, and the validator and grower simulate the interaction of the user with the application. All three steps were closely modeled according to the real application used in MC20 [22]. Just as in the real application, data were passed back and forth between the components, alternating between clustering, growing and validation. Throughout the process, each object received a cluster label and potentially a set of non-matching (“rejected”) cluster labels. The number of virtual clicks was recorded. Details are given in Appendix A.

In the validation step, the clusters are evaluated similar to what a human user would do: clusters are accepted if their purity is above the validation threshold $t_{v}\in \left(0,1\right)$, and heterogeneous clusters are deleted. The purity of a cluster was calculated as the fraction of objects matching the cluster’s majority gold standard label. If a cluster is accepted, all objects with a non-matching gold standard label are removed from this cluster. For each rejected object, we saved the non-matching cluster label. This information is therefore available to improve the clustering and growing in future iterations. In the subsequent growing step, each surviving cluster was revisited in order to determine the proper, larger, cluster boundaries. All thus far unclustered objects, ordered by increasing distance to the cluster centroid, formed the cluster candidates. These cluster candidates were treated in batches. The batch size was hard-coded in the MorphoCluster application as 50. The batches were visited using exponential search [27], an algorithm for searching an unbounded key space: the first batch of non-fitting images was found by jumping forward with increasingly larger steps. Subsequently, binary search was used to examine the range between the last accepted (fitting) batch and the first rejected (non-fitting) batch. If the purity of a batch is below the unpure batch threshold $t_{gu}\in \left(0,1\right)$, the batch is rejected and the search interval shrinks. If the purity is between $t_{gu}$ and the pure batch threshold $t_{gp}\in \left(0,1\right),t_{gu}\leq t_{gp}$, individual non-matching objects are removed and a linear search mode is entered. Usually, many pure batches can be added before the first impurities arise. The objects found to be similar are added to the cluster, and the others are returned to the pool of unclustered objects. As in MC20, we recalculated the cluster centroid after a number of objects were added and restarted the growing step.

After validation and growing, a new iteration begins: the remaining unclustered objects are clustered and the new clusters are validated and grown. This was repeated until a maximum number of iterations or until a certain threshold of objects assigned to a cluster was met. In a real application scenario, the resulting clusters would then be arranged in a hierarchy and a user would assign meaningful names to provide a taxonomic annotation of the complete dataset.

We set the validation threshold to $t_{v}=0.85$, the pure batch threshold $t_{gp}=0.75$ and unpure batch threshold $t_{gu}=0.55$. Details on how these values were chosen can be found in Appendix A.

### 2.2. Representation Learning and Feature Extraction for MorphoCluster

Any deep image recognition model can be used for feature extraction by removing all training-specific layers and using the backbone to calculate image representations [28] that are suitable for distinguishing between known and new categories beyond the categories used in the training set. The distance in the representation space between two objects then serves as a proxy for their similarity [22].

#### 2.2.1. Representation Learning

In MC20 [22], a deep learning feature extractor based on a ResNet18 [29] classifier was trained on UVP5/EcoTaxa, which is a coarsely annotated subset of UVP5/MorphoCluster [30]. We call such a supervised representation learning approach that is enabled through target domain labels non-blind. In this work, we additionally used ZooScanNet [31] as a source dataset. For model training, we used a learning rate of 1 × 10^−4^, a weight decay of 0.003, cross-entropy loss, undersampling to up to 1000 objects per class and no sample reweighting. Details on how these values were chosen can be found in Appendix B.1.

However, since the primary purpose of MorphoCluster is the exploration and annotation of completely new data, it can also be necessary to train a feature extractor without prior label information from the same imaging modality. This might be the case if a new imaging instrument is deployed and the recorded images are annotated for the first time.

When the target dataset is sufficiently similar to a labeled source dataset, representation learning can be implemented through transfer learning [32,33] by training a feature extractor supervised on labeled source data stemming from a similar imaging modality (e.g., as suggested by Orenstein and Beijbom [34]). Here, we used feature extractors trained with the labeled data of the UVP5/EcoTaxa [30] and ZooScanNet [31] datasets, as well as a pretrained classifier model based on the ImageNet dataset [35], to calculate image representations for the remaining other plankton image datasets.

If no sufficiently similar labeled data are available, unsupervised learning can be employed, where a feature extractor is trained on the target data alone with no further information apart from the images themselves [36]. To investigate whether useful image representations can be learned without label supervision, we therefore also trained the same deep learning feature extractor as in supervised learning using the momentum contrast (MoCo) approach by He et al. [37] (refined as MoCo v2 by Chen et al. [38]). This self-supervised technique generates two different views of an input image using data augmentation, which are fed through a query encoder and a momentum encoder network, respectively. The MoCo loss is formulated as an instance discrimination task, where a model is trained to discriminate between individual object instances [39]. This loss ensures that both versions of the same image receive similar representations while being dissimilar to all previously seen images. We used a momentum of 0.99, a weight decay of 0.0001, a learning rate of 1 × 10^−4^ and temperature τ=0.07. Details on how these values were chosen can be found in Appendix B.3.

We called transfer learning and unsupervised learning blind, as target domain labels were not used.

All used models were based on the ResNet18 architecture [29], as this architecture proved to be computationally efficient and still sufficiently discriminative [40], and produced 512-dimensional image representations. During training, the validation loss was observed and early stopping was used to avoid overfitting. The Adam algorithm [41] was used to optimize the model parameters. The training data were augmented using random geometric and photometric distortions (rotate, flip, affine projection, blur, intensity change, contrast change) that were applied to the training images. The models were trained with PyTorch [42] on a NVIDIA GeForce GTX TITAN X or GeForce RTX 2080 Ti GPU using cosine learning rate decay [43] and a batch size of 1024 for up to 500 epochs.The parameters of the model backbones were initialized using publicly available ImageNet-trained model parameters.

#### 2.2.2. Dimensionality Reduction for Image Representations

There are two processes that are time-demanding in the MorphoCluster process: the clustering and the distance calculations in the growing step. As the growing step is interactive, these calculations should be as fast as possible to avoid unnecessary waiting time for the user. The runtime of the clustering and the distance calculations strongly depend on the dimensionality of the image representations. Therefore, a small number of dimensions is essential. However, the representation still needs to encode enough information to enable accurate clustering and similarity search. Dimensionality reduction methods serve to reduce the dimensionality of image features while preserving their expressiveness. In this work, we compared different methods of dimensionality reduction to find the most suitable approach for MorphoCluster.

In MC20, a multi-stage trained projection layer was used to reduce the dimensionality of features to d=32 as a trade-off between the amount of encoded information, the size in memory and the runtime of clustering and distance calculations [22].

We studied three different types of dimensionality reduction in this work:

- single-stage: The projection layer was trained together with the feature extractor in one pass;
- multi-stage: First, the feature extractor model is trained as-is. Then, the projection layer is inserted and the complete model is fine-tuned;
- pca: The feature extractor is trained in the usual way and used to calculate features of full dimensionality for the images of the target dataset. The dimensionality of these features is then reduced using principal component analysis (PCA).

The projection layer used in the single-stage and multi-stagecondition consisted of a ReLU nonlinearity and a *d*-dimensional linear layer, which were appended to the ResNet18 backbone.

We kept d=32 and trained the single-stage model for 500 epochs with a learning rate of 1 × 10^−4^, and the second stage in the multi-stage condition for only 250 epochs with a lower learning rate of 1 × 10^−5^. These settings are motivated in Appendix B.2.

### 2.3. Clustering Algorithms for MorphoCluster

MorphoCluster assumes very pure clusters in the validation step which requires a clustering algorithm that labels only the densest regions as clusters and leaves less certain objects unlabeled.Moreover, the clusterer needs to support large datasets, as typical plankton image datasets can contain millions of images.

In MC20, the density-based clustering algorithm HDBSCAN* [44] was used [22]. It satisfies these requirements, and the leaf cluster selection method produces fine-grained, homogeneous clusters, but the choice of its parameters is not straightforward. The prototype-based *k*-means, on the other hand, is a well known clustering algorithm with only a single intuitive parameter. It is used in many areas [45] and scales well with dataset size, can cluster even billions of samples and a wide range of implementations exist for different architectures [46,47]. However, like most clustering algorithms, *k*-means partitions the dataset, i.e., every object is assigned to a cluster. This would render the separation of validation and growing, a core component of MorphoCluster, impossible.

To exploit the advantages of *k*-means and still meet the requirements of MorphoCluster, we here propose two extensions that are tailored specifically for the MorphoCluster approach: shrunken *k*-means (S-*k*-means) and partially labeled *k*-means (PL-*k*-means).

#### 2.3.1. Shrunken *k*-Means

We devised shrunken *k*-means (S-*k*-means) as a a modified version of regular *k*-means that reduces the computed clusters to their very core, subsequent to the actual clustering step: First, regular *k*-means was used to partition the dataset into distinct clusters. Afterwards, the distance of every object to its respective cluster centroid was calculated. Finally, the previously calculated cluster labels were only retained for a small quantile $p_{\mathrm{core}}$ of objects that are closest to their respective centroid in each calculated cluster. The remaining objects were un-assigned and returned to the pool of unclustered objects. For $p_{\mathrm{core}}=1.0$, shrunken *k*-means is identical regular *k*-means. The exact algorithm is given in Appendix C.

#### 2.3.2. Partially Labeled *k*-Means

Most clustering algorithms are meant to be applied once to the whole dataset. In contrast, MorphoCluster involves the repeated application of clustering interlaced with the validation and cleaning of the resulting clusters until all objects are treated [22]. Therefore, a clustering run in a later iteration could potentially benefit from the previous annotations. Moreover, so-far unclustered objects that were missed in previous growing steps could still belong to a nearby existing cluster.

We further extended the shrunken *k*-means clustering algorithm to incorporate the already validated clusters into the cluster formation. Here, they can guide the discovery of more meaningful clusters and allow for the late assignment of so-far unclustered objects. For that, we modified both the expectation and the maximization step of the *k*-means algorithm. We called this modification partially labeled *k*-means (PL-*k*-means) [48].

In contrast to regular *k*-means or other clustering algorithms, we explicitly used the positive and negative feedback obtained in the validation and grow steps. This information is encoded in the Boolean rejection matrix $R\in B^{n\times k}$ (with k∈N being the number of clusters and n∈N the number of objects):

(1) $$R_{ij}=\left\{\begin{array}{cc} \mathrm{True} & \mathrm{if}\;\mathrm{sample}\;i\;\mathrm{was}\;\mathrm{rejected}\;\mathrm{for}\;\mathrm{cluster}\;j\\ \mathrm{False} & \mathrm{otherwise} \end{array}\right.$$

If a sample *i* is ultimately assigned to a cluster, the row $C_{i}$ contains only one false entry.

The rejection matrix $R_{ij}$ was then used to steer the label assignment (expectation step). Due to the fact that *k*-means clustering is usually highly sensitive to noise, we introduced a noise fraction $p_{\mathrm{noise}}$ that makes the re-calculation of the cluster centers (maximization step) more robust by using only objects close to the previous centers. Otherwise, the algorithm is very similar to regular *k*-means. In the end, the same cluster shrinking step as in shrunken *k*-means was performed. The exact algorithm is given in Appendix D.

In each iteration, we increased the number of clusters by the cluster count increment $k_{\Delta }$ so that more and more smaller and smaller clusters were found over time.

### 2.4. Evaluation of the Proposed Methods

#### 2.4.1. Evaluation of Feature Extractors

Commonly, the performance of a feature extractor is measured by the performance of the respective downstream task. As a result of the complexity of the respective downstream tasks (here, the simulation of the MorphoCluster approach), the accuracy of a classifier [37,49,50] or supervised cluster evaluation scores [51,52] are often used. Here, we used the F1 score of a nearest centroid classifier [53] trained on a held-out test set as an indicator of the expected performance.

##### F1 Score of a Nearest Centroid Classifier (NCC-F1)

Whenever target labels were available for the evaluation, we used the the F1 score of a nearest centroid classifier (short NCC-F1) to quantify how compact and well-separated the classes are in the representation space. To evaluate the representations of a certain set of images, a nearest centroid classifier was fitted to these representations. The fitting error was measured using the F1 score. The score ranged from 0 to 1 and was used as an indicator of how well the representations satisfied the premise of dense, well-separated, spherical clusters with equal variance. These properties will facilitate the later unsupervised partition of the representation space into visually distinct and homogeneous clusters. The NCC-F1 was used for supervised model selection and final model comparison.

#### 2.4.2. Evaluation of Clustering Algorithms

We compared the fitness of our novel clustering algorithms to the established HDBSCAN* using our simulation of the MorphoCluster annotation process, optimizing the following antagonistic performance metrics:

- *Efficiency:* The primary goal is to maximize the efficiency of the annotation. In this context, we define efficiency as the number of objects sorted per virtual click;
- *Completeness:* The more objects that are annotated, the more inefficient the process becomes. At some point, the remaining objects cannot be grouped into valid clusters any more, and the process is stopped. We define completeness as the fraction of objects that can be handled efficiently;
- *Precision:* By hiding most individual objects from the user using exponential search, MorphoCluster trades a bit of precision for more efficiency. Nevertheless, high precision is the goal of every image annotation approach. Here, we define precision as the mean precision over all clusters measured by their object’s gold standard label;
- *Final Number of Clusters:* After a dataset has been partitioned into homogeneous clusters, these have to be given taxonomic labels. Many small clusters can lead to a high precision but are more tedious to label afterwards, negating the accelerating effect of the MorphoCluster approach. We therefore strive to minimize the final number of clusters (#Clusters) after partitioning.

### 2.5. Datasets

**Figure 2:**
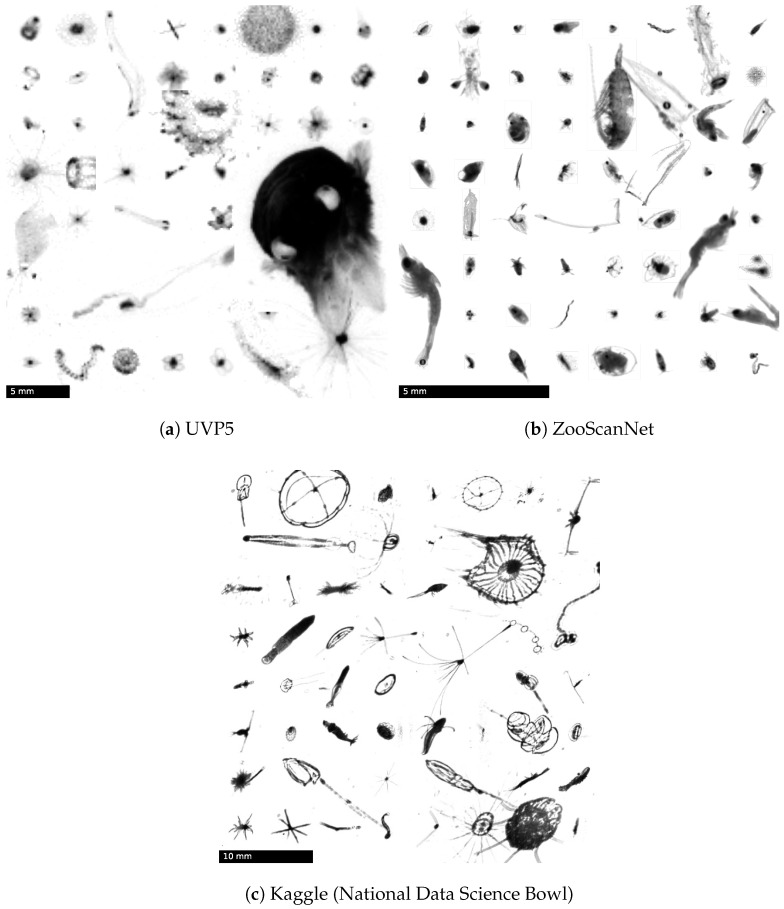
Mosaics of plankton image datasets. Objects were randomly sampled from the datasets and arranged in an aesthetically pleasing manner. UVP5 (**a**) © Kiko and Schröder 2020, CC BY-NC [30]. ZooScanNet (**b**) © Elineau et al.2020, CC BY-NC [31]. Kaggle (**c**) © Cowen, Sponaugle, Robinson and Luo, Oregon State University, 2015 [56]. Reproduced with permission from the authors.

We used four plankton image datasets to evaluate the different representation learning and clustering approaches. UVP5 [30] (Figure 2a) consists of images taken by the Underwater Vision Profiler 5 (UVP5) [9] in the pelagic zone of various oceans.Two labelings are available: The original labeling of the data, UVP5/EcoTaxa, contains 96k labeled images in 65 categories. UVP5/MorphoCluster is the result of the first application of MorphoCluster (MC20) [22] and contains 1.2 M images in 280 categories.

ZooScanNet [31] (Figure 2b) consists of 1.4 M images in 93 categories acquired with the ZooScan system [10], a customized flatbed scanner with a transparency unit for digitizing preserved wet net samples.

WHOI-Plankton [54] comprises 330 k labeled images in 103 categories, taken in 2014 by Imaging FlowCytobot (IFCB) [55], a submersible imaging flow cytometer.

Kaggle (official title: PlanktonSet 1.0) [56] (Figure 2c) is part of the National Data Science Bowl competition hosted on Kaggle [57]. It contains approximately 30 k images in 121 categories. The images were recorded using the In Situ Ichthyoplankton Imaging System (ISIIS) [58], a submersible shadowgraph-based imaging system that produces kilometer-long continuous images as its line scan camera moves through the water.

ImageNet [35] is a widely used dataset to train deep learning models. Its training split consists of 1.2 M images in 1000 categories. The images are color photographs of every-day objects [59] and therefore very dissimilar to the grayscale microscopy-type images in the plankton image datasets.

## 3. Results

Within this section, we first compare the simulation performance to the manual experiment in MC20 [22] (Section 3.1). Next, results of the application of different feature extractors and methods of dimensionality reduction to multiple plankton datasets are shown in Section 3.2. The three clustering algorithms under investigation are then compared with respect to efficiency, completeness, precision and the total number of clusters, using the simulation in Section 3.3. Finally, the top performing representation learning and clustering approaches are combined in a final comparison (Section 3.4). All experiments were organized using experitur [60].

### 3.1. Simulation Performance

To ensure that the simulation adequately approximates the user behavior, we replicated the manual experiment from MC20 [22] in our simulation using the same objects, feature vectors and clustering hyperparameters. The UVP5/MorphoCluster labels [30] produced in MC20 were used to decide the cluster membership.

**Figure 3:**
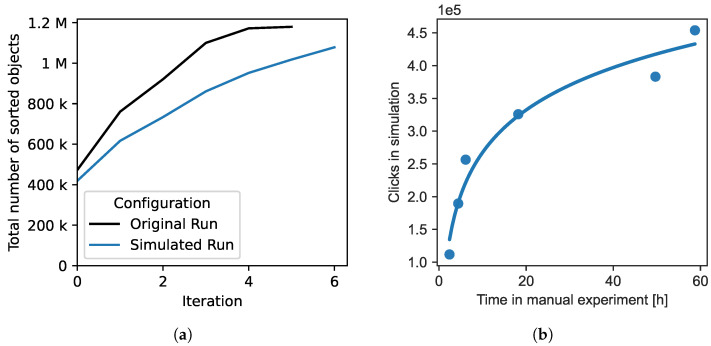
Comparison of original and simulated annotation, based on the UVP5/MorphoCluster labels generated in MC20. (**a**) Total number of sorted objects; (**b**) Correspondence between number of simulated clicks and time required for manual operation.

Figure 3a compares the sorting progress of the manual and the simulated run in each step. The simulation requires an additional step because fewer objects are sorted in the earlier steps, and so the simulation does not reach full completeness. However, while more objects could be annotated in each step in the manual experiment, the plots of the simulation and manual run display a similar behavior, which is steeper at the beginning with increasing saturation towards the end. As Figure 3b shows, the number of simulated clicks is a monotonic function of the time required for manual operation. In conclusion, the simulated behavior is, in our view, similar enough to serve as a basis for the comparison of representation learning approaches and clustering algorithms in the following experiments.

### 3.2. Representation Learning

To further evaluate the choices made in MC20 and to identify the best representation learning approach, we trained different feature extractors using the images and labels from UVP5 and ZooScanNet datasets and compared them using the NCC-F1 score (see Section 2.4 for details). This includes supervised, transfer and unsupervised approaches. We also investigated different approaches to dimensionality reduction.

In Table 1, we provide the test NCC-F1 score for the three different methods of dimensionality reduction to d=32 and compare them to a baseline without dimensionality reduction (full). One feature extractor was trained for every source dataset and the dimensionality reduction method and features are calculated and evaluated for four target plankton datasets: ZooScanNet, UVP5/EcoTaxa, WHOI and Kaggle. Additionally, we include the results of the unsupervised training of the UVP5 and ZooScanNet models.

Test NCC-F1 was calculated on the test split of the respective test dataset after dimensionality reduction.

In the case of the UVP5/EcoTaxa dataset, all scores are comparatively low (below 40%), even when using the full 512 feature dimensions. This could be explained with the low sorting precision of this dataset, which is also noted in MC20 [22].

All reduced methods produce results that are nearly as good as full dimensionality. This suggests that even only 32 dimensions encode enough information to successfully classify an image.

#### 3.2.1. Availability of Target Labels

For any given target dataset, when trained on labeled target domain data (non-blind), the feature extractor consistently performs better than when labeled data are not available (blind). This is to be expected as the image labels contain valuable information about meaningful divisions of the representation space.

#### 3.2.2. Transfer vs. Unsupervised Learning

Independent of the dimensionality of the features (full and pca), unsupervised learning (unsup) on the target dataset performs slightly better than the respective best transfer learning approach (ImageNet feature extractor followed by PCA dimensionality reduction). However, we only observe a small performance gap in this experiment.

#### 3.2.3. Source Dataset in Transfer Learning

ImageNet outperforms the plankton datasets as a source for any transfer learning task, despite being less similar to the target data. For the WHOI and Kaggle targets, the second best sources are UVP5/EcoTaxa and ZooScanNet, respectively, so neither of them are inherently better for knowledge transfer.

#### 3.2.4. Dimensionality Reduction Methods

When training data for the target instrument were not available (blind), PCA was the only unsupervised option that we explored. For transfer learning, single-stage and multi-stage, where the dimensionality reduction is trained using a source dataset with a different distribution, do not perform as well.

On the other hand, when training data are available for the target dataset (non-blind), a trained projection layer has a slight advantage over PCA dimensionality reduction. For UVP5/EcoTaxa, single-stage performs slightly better, and for ZooScanNet, multi-stage has an advantage. However, both are in the same order of magnitude as pca, and the small performance gains are offset by disproportionately more complex training.

### 3.3. Comparison of Clustering Algorithms

In the following section, we compare Python implementations of HDBSCAN* [61] and the proposed shrunken *k*-means and partially labeled *k*-means clustering algorithms using the simulation framework, the UVP5/MorphoCluster and ZooScaNet datasets and the original features from MC20  [22].

We set k=8 and $m_{0}=128$ for HDBSCAN* (deviating from MC20); k=1000 and $p_{\mathrm{core}}=0.01$ for shrunken *k*-means; and $p_{\mathrm{noise}}=0.1$, $k_{\Delta }=100$, $k_{0}=500$ and $p_{\mathrm{core}}=0.01$ for PL-*k*-means. These choices are motivated in Appendix E.

In Table 2, we compare the performances of HDBSCAN*, S-*k*-means and PL-*k*-means using the UVP5/MC and ZooScanNet datasets with the original HDBSCAN* configuration with k=1 [22].

While the original HDBSCAN* configuration yields the highest completeness, it also entails the lowest sorting efficiency and a high number of resulting clusters, which made the final naming step in MC20 very time-demanding [22]. It is, therefore, excluded from the following analysis.

The highest completeness is achieved using PL-*k*-means, whereas HDBSCAN* and S-*k*-means perform slightly worse. The number of clusters is acceptable for all three clustering algorithms, whereas the lowest numbers are obtained using HDBSCAN* and S-*k*-means. All clustering algorithms lead to a high sorting efficiency, with 6 to 12 objects sorted with every click. The best efficiency is provided by S-*k*-means, while HDBSCAN* and PL-*k*-means perform very similarly. The precision is generally high and does not differ substantially between the different clustering algorithms. HDBSCAN* stands out by taking by far the least total time to complete the experiment in both configurations, whereas PL-*k*-means takes the longest.

In summary, no one clustering algorithm is the best in terms of all performance metrics.

### 3.4. Comparison of Feature Extractors for Clustering

In the previous experiments, we compared different feature extractor techniques using the NCC-F1 score and also assessed the performance of the three proposed clustering techniques using the MorphoCluster simulation. Now, we assess the performance of the feature extractors using the best performing clusterer (HDBSCAN*) in the MorphoCluster simulation. For the UVP5/MorphoCluster and ZooScanNet target datasets, we used the respective blind and non-blind representation learning techniques. A fully supervised feature extractor, where the training dataset is identical to the target dataset, is included to estimate an upper performance bound. Image representations were calculated for the respective target dataset and their dimensionality was reduced to d=32 with PCA. The respective representations were then used in the simulation of the MorphoCluster process with the previously selected configuration of HDBSCAN*.

The results are summarized in Table 3. All three representation learning approaches generate an acceptable number of clusters that enables quick cluster naming in the final step. Independent of the representation learning approach and dataset, the precision is well over 95%.

For both target datasets, supervised training, where labeled training data for the target domain are available, leads to the highest completeness and efficiency. This was to be expected because the feature extractor is taught the exact information that is required in the evaluation, i.e., the correspondence of certain morphologies and labels.

When the source and target dataset are the same (fully supervised), the obtained scores are an upper limit for the real performance. In the real application, exactly the same data that were used to train the feature extractor would usually not be labeled again. However, often new data from an already labeled domain need to be annotated. Here, the annotation of this new data would benefit from a feature extractor trained supervised with the existing labeled data. Depending on the distribution difference between labeled and new data, the annotation performance could approach the upper limit established here.

In the blind conditions where labeled training data for the target domain are not available, large completeness gains can be realized by using unsupervised learning instead of transfer learning. In terms of efficiency, both approaches are very similar. The higher number of clusters with unsupervised learning is a hint that an unsupervised-learned representation space is less well-aligned with the concepts used for annotation.

For the UVP5/MC target dataset, even when trained fully supervised, the completeness is fairly small in comparison to Table 2. There, the image representations used for clustering were already used in the first MorphoCluster run (MC20 [22]) to create the labels, whereas, here, new feature extractors have been trained.

## 4. Discussion

Despite the dire need for efficient tools to annotate large amounts of image data in many fields [1,2,3,4,5,6], a formal exploration of different image annotation approaches is sparse. A formal comparison of different approaches would require additional setup to collect the required metrics and significant effort to annotate the same data multiple times. New approaches are therefore often only supplemented with anecdotal data [20,22,62,63,64] and many tools are published without a formal analysis. For MorphoCluster, we here ameliorate this problem with a simulation. A similar simulation has been used by Cohn et al.to test their semi-supervised clustering algorithm for the annotation of news paper articles [65].

### 4.1. Simulation of the Manual Annotation Process

Our threshold-based simulation approach does not model the complex behavior of a human user in all detail. However, we were able to simulate the central clustering and annotation phase of MorphoCluster accurately enough to qualitatively benchmark modifications to the initial MorphoCluster setup objectively and without having to invest hundreds of work hours into new manual annotation trials and the implementation of new functionalities in the web application. In our view, it is, therefore, a powerful tool that allowed us to thoroughly test many different representation learning and clustering configurations.

### 4.2. Representation Learning

As an explorative image annotation method, MorphoCluster is usually applied when labels for the target dataset are insufficient or not available. In this work, we analyzed different methods of representation learning when labeled data from the target domain are available (non-blind) or not available (blind).

Early approaches to image annotation rely solely on handcrafted image features [10,66] that require no training. More recently, classifier-based [34,67] or contrastive [62] approaches were successfully used for the supervised deep representation learning in the field of biological oceanography. The classifier-based approach is often easier to apply in practice, as contrastive approaches tend to require larger computational resources [39,68,69] and elaborate sample mining strategies [70]. Schmarje et al.give an overview on representation learning in a more general context [36].

We conducted a series of experiments to provide guidance on the selection of a method in each condition, which will be useful for image annotation using the MorphoCluster approach. Unsurprisingly, if labeled data from the target domain are available (non-blind), the best option is to use these data for supervised training. If no labeled data from the target domain are available (blind), the unsupervised MoCo v2 [38] feature extractor performed best in our experiments. Other popular unsupervised representation learning approaches include SimSiam [71], SimCLR [69] or BYOL [72]. These, however, require much larger batch sizes, display a smaller accuracy or had no public implementation available that fits into our experimental framework, and were therefore not used.

Supervised and unsupervised representation learning approaches entail a deep learning training phase. In the case of scarce computational resources, we find that transfer learning using a generic ImageNet-trained feature-extractor is a good solution as it outperforms the plankton datasets as a source in this case. One explanation for this comparatively good performance could be that the number of categories (1000) is much larger compared to the plankton datasets (65 to 121 categories) and that ImageNet is more curated and therefore contains few ambiguous images.

Since the projection layer for single-stage and multi-stage dimensionality reduction is trained, it is conditioned on the *training dataset* and therefore cannot adapt to the dataset shift. Meanwhile, pca equally retains the expressiveness of image representations, but is conditioned on the target dataset and is therefore more flexible and robust to distribution shifts, which regularly occur, even without changing the instrument. Therefore, it is advisable to use PCA dimensionality reduction in both blind and non-blind situations.

In the final comparison (Section 3.4), all representation learning methods yield a fairly small completeness for the UVP5/MC target dataset. This can be explained by the fact that the MorphoCluster labeling of the UVP5 dataset is only one of possibly many valid labelings and that the simulation treats interchangeable classes as distinct. Just by training another model on the same data, a different representation space was learned that is less aligned with the UVP5/MorphoCluster labels.

### 4.3. Clustering

In MC20, we selected the density-based HDBSCAN* clustering algorithm to generate candidate clusters for annotation [22]. Density-based approaches come with a range of favorable properties, e.g., the detection of clusters of differing density and arbitrary shape or noise filtering [73]. However, few Python implementations exist, and, in preliminary experiments, only HDBSCAN* was able to deal with the size of the used plankton datasets.

Here, we devised two alternatives specifically tailored to the requirements of MorphoCluster, S-*k*-means and PL-*k*-means.

The idea of modifying *k*-means so that it allows samples to be classified as noise is not new. NK-means [74] is a preprocessing step for *k*-means to remove outliers from a dataset prior to clustering. However, this operation is prohibitively expensive for the size of the used datasets. Similar to our S-*k*-means and PL-*k*-means, KMOR [75], *k*-means- [76] and the partial clustering algorithm by Tian et al. [5] exclude objects with a large distance to their nearest center. However, these algorithms are more complex, which prevents their use with large data sets, or their parameters are hard to tune, which makes their thorough experimental exploration very costly. Our S-*k*-means and PL-*k*-means algorithms differ from these previous works by only retaining a configurable fraction of objects $p_{\mathrm{core}}$ from each individual cluster after clustering. Furthermore, PL-*k*-means excludes a fraction of objects $p_{\mathrm{noise}}$ from the recalculation of the cluster centers. In our experiments, excluding outliers after clustering ($p_{\mathrm{core}}<1.0$) was beneficial for efficiency, whereas modeling noise ($p_{\mathrm{noise}}>0$) had no substantial influence on the target metrics.

Our PL-*k*-means also bears resemblance to constrained-*k*-means in that it constrains the objects with a known label to stay in the corresponding cluster [77]. However, going beyond this algorithm, PL-*k*-means allows positive information about an object (“object is in class X”), as well as negative information (“object is not in class Y”), which is naturally generated in the validation and growing steps. Other clustering algorithms constrain the outcome using must-link and cannot-link constraints between objects [65,78].

Plankton image data are usually accompanied by environmental and spatio-temporal metadata, and it was shown that these metadata can improve the classification of plankton images [79]. Therefore, it could be interesting to include these additional data in the clustering and annotation process to identify patterns beyond the purely visual. To reconcile the different modalities of deep image representations and environmental and spatio-temporal metadata, multi-view clustering methods might be necessary [80,81].

We found that, overall, the tested algorithms perform comparably, and any of the three can be used. The newly developed clustering algorithms S-*k*-means and PL-*k*-means are advantageous if the completeness is more important than the efficiency or runtime, or for smaller datasets. HDBSCAN*, on the other hand, runs much faster than its competitors and therefore might be better suited for real-world applications. However, being based on *k*-means, S-*k*-means and PL-*k*-means inherit its potential for more efficient implementations in the future.

In contrast to MC20, we found that, for HDBSCAN*, the initial minimum cluster size $m_{0}$ is of lesser importance than previously assumed and that, instead, the neighborhood size *k* should be increased above the previous choice to k=8.

### 4.4. Practicality of the Results

The primary goal of MorphoCluster is the maximization of annotation efficiency to keep up with the constant inflow of new image data. In our experiments, this value was often higher than five objects per click. The best efficiency was obtained with supervised representation learning and S-*k*-means (8–12 objects per click).

Annotation completeness, the percentage of the dataset that could be efficiently sorted, is important, as a high completeness reduces the amount of data that need to be post-processed using different annotation approaches. According to our results, the completeness mainly depends on the training mode of the feature extractor. If labeled target data were available, up to 84% of a dataset could be sorted with high efficiency (ZooScanNet, fully supervised). Otherwise, a completeness of up to 57% was achieved. However, it is important to note that the performance of the simulation used here is not indicative of the performance of a human operator, who could possibly achieve a much higher completeness. The simulation results only enable a comparison of different configurations. The simulation treats objects as outliers, even if their given label could be plausibly interchanged with the cluster’s majority label (e.g., in the case of sub-classes), and stops the annotation. In a real-world annotation, however, it is very likely that a user accepts these plausible members, and we therefore expect that a much larger completeness can be achieved. In fact, the UVP5/MorphoCluster labeling was annotated in MC20 using features trained on the same UVP5/EcoTaxa labeling used here, and a high completeness of 98.63% was achieved [22].

In this work, we take the annotations provided with the used datasets not as ground truth but as the annotations of just another expert. The annotation precision we measure here is, therefore, actually the consistency with these gold standard labels. It reaches or even surpasses the self-consistency levels experts commonly reach under optimal circumstances [82] (95% to 99%) for all image representations, clustering configurations and datasets. Moreover, plankton image annotation is particularly difficult, as the captured data provide only a very limited view of the physical world and lacks spatial and temporal aspects and often even color, not to mention non-visual qualities. Therefore, image annotation is a priori always subject to uncertainty and interpretation. In Appendix F, we show the outliers of two gold standard classes as an illustration. Accordingly, the small variations in the throughout high precision are less significant than completeness or efficiency, and we conclude that MorphoCluster is able to approximate the true annotation of a dataset with a high consistency in most scenarios. Like in the real MorphoCluster application, the simulation reaches this high annotation precision while also investigating only a reduced number of images for growing. This is a strong indication that the MorphoCluster approach of clustering, validation and growing can, in comparison to conventional one-by-one sorting, save manual effort by approximating the true annotations with a reduced number of images a user has to review without sacrificing precision.

## 5. Conclusions and Outlook

In 2020, we presented MorphoCluster, a cluster-based alternative to the thus far prevalent classifier-based approach to image annotation (MC20) [22]. In this work, we provide the first simulation-based verification of the MorphoCluster image annotation paradigm.

Our contributions comprise two new special-purpose clustering algorithms and a detailed analysis of different clustering and representation learning scenarios using a simulation of the MorphoCluster approach. We were able to improve upon the algorithmic choices made in MC20 and we acquired results that can motivate further research for the optimization of MorphoCluster.

As a result of this work, we recommend using supervised (if any labeled data are already available) or unsupervised (if no labeled data are available) representation learning and HDBSCAN* clustering with a neighborhood size of k=8 and an exponentially decaying minimum cluster size. These settings will be incorporated into a future version of the MorphoCluster image annotation application.

We would also like to note that the simulation developed here could be integrated into the MorphoCluster process as a fully automated pre-processing step to filter out simple, very homogeneous and well known classes. By detecting dense regions in the representation space of a target dataset merged with a labeled training dataset and assigning the respective majority training label (if present), the speed of MorphoCluster could be further increased. The common problems of classifier-based approaches [22], where outliers and objects from previously un-annotated classes are forced into the next best class, could probably be avoided. Such data would instead remain unlabeled, whereas common objects would be labeled automatically with high confidence. Distribution differences between training and new data would not affect the annotation, and training data biases would not be carried over into the newly annotated data.

## Figures and Tables

**Table 1:** Quality of supervised and unsupervised image representations measured as the test NCC-F1 on each target dataset for each condition and source dataset, respectively. The first rows show the test scores for full feature dimensionality for reference, the following rows show the results for different methods of dimensionality reduction to d=32. All models were trained supervised, unless unsupervised training is indicated by ^unsup^. Conditions where labeled target domain data were used for training (non-blind) are underlined. The best blind and non-blind result is bold, respectively, the second best is italic.

Dim. Red.	Target →	UVP5/EcoTaxa	ZooScanNet	WHOI	Kaggle
	Source ↓				
full	ImageNet	13.44%	21.40%	34.66%	38.99%
	UVP5/EcoTaxa	38.03%	18.18%	15.54%	30.46%
	ZooScanNet	11.52%	62.51%	17.02%	25.24%
	UVP5/EcoTaxa^unsup^	13.94%			
	ZooScanNet^unsup^		21.56%		
pca	ImageNet	*11.43%*	*18.47%*	**23.09%**	**33.16%**
	UVP5/EcoTaxa	33.18%	16.09%	12.29%	*27.33%*
	ZooScanNet	10.01%	58.97%	*13.78%*	21.86%
	UVP5/EcoTaxa^unsup^	**12.73%**			
	ZooScanNet^unsup^		**20.21%**		
single-stage	UVP5/EcoTaxa	**37.91%**	14.22%	10.55%	24.22%
	ZooScanNet	9.75%	*62.44%*	10.20%	20.34%
multi-stage	UVP5/EcoTaxa	*37.58%*	13.17%	9.94%	22.57%
	ZooScanNet	9.07%	**63.09%**	13.09%	15.96%

**Table 2:** Comparison of clustering algorithms regarding completeness, number of resulting clusters, efficiency, sorting precision and clustering runtime. The best result for each respective metric is bold, the second best is italic.

Clustering Algorithm	Dataset	Completeness ↑	#Clusters ↓	Efficiency ↑	Precision ↑	Runtime ↓
HDBSCAN* (k=1)	UVP5/MC	91.53%	19,047	3.35	99.70%	6.84 h
	ZooScanNet	93.66%	17,908	4.42	99.86%	4.25 h
HDBSCAN* (k=8)	UVP5/MC	*80.51%*	1563	*6.63*	97.07%	**2.67 h**
	ZooScanNet	83.98%	**914**	9.77	97.97%	**1.56 h**
S-*k*-means	UVP5/MC	77.01%	**862**	**7.81**	95.75%	*15.52 h*
	ZooScanNet	*85.01%*	*947*	**11.52**	98.15%	*22.86 h*
PL-*k*-means	UVP5/MC	**85.66%**	*927*	6.36	95.92%	104.11 h
	ZooScanNet	**88.67%**	1482	*9.95*	98.86%	134.97 h

**Table 3:** Comparison of feature extractors regarding completeness, number of resulting clusters, efficiency, sorting precision and clustering runtime when using the previously selected configuration of HDBSCAN*. The best result for each respective target dataset is bold, the second best is italic.

Training	Target Dataset	Completeness ↑	#Clusters ↓	Efficiency ↑	Precision ↑
Transfer (ImageNet)	UVP5/MC	14.60%	**1148**	2.94	98.85%
Unsupervised (UVP5/MC)	UVP5/MC	19.13%	4509	2.22	99.49%
Supervised (UVP5/ET)	UVP5/MC	*27.57%*	*1265*	**4.03**	99.07%
Fully Supervised (UVP5/MC)	UVP5/MC	**44.07%**	3329	*3.99*	99.46%
Transfer (ImageNet)	ZooScanNet	39.33%	*1397*	4.24	97.39%
Unsupervised (ZooScanNet)	ZooScanNet	*57.31%*	2866	*4.39*	97.72%
Fully Supervised (ZooScanNet)	ZooScanNet	**83.98%**	**914**	**9.77**	97.97%

**Figure A1:**
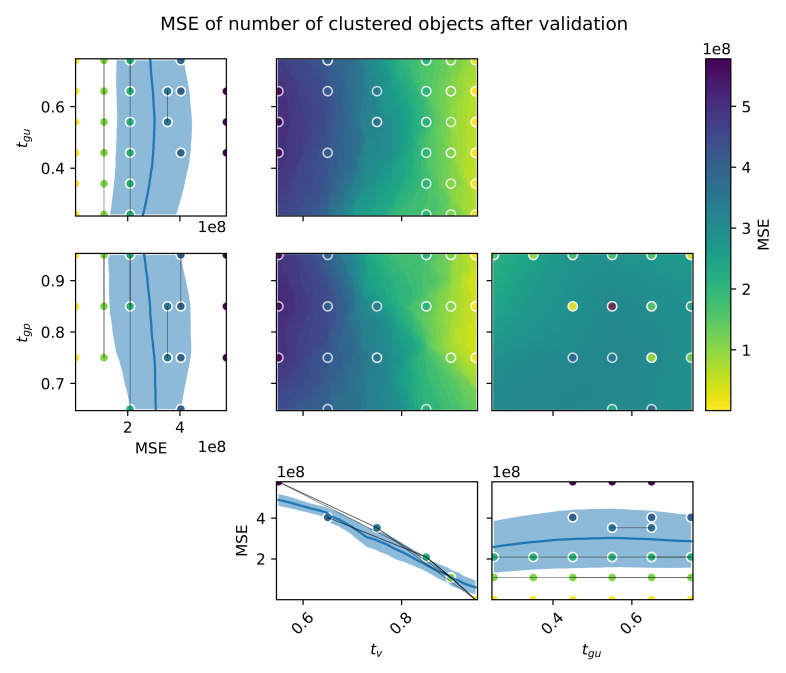
MSE of the number of validated objects as a function of the simulation hyperparameters after one step of the simulation. The larger the validation threshold $t_{v}$, the smaller the discrepancy.

**Figure A2:**
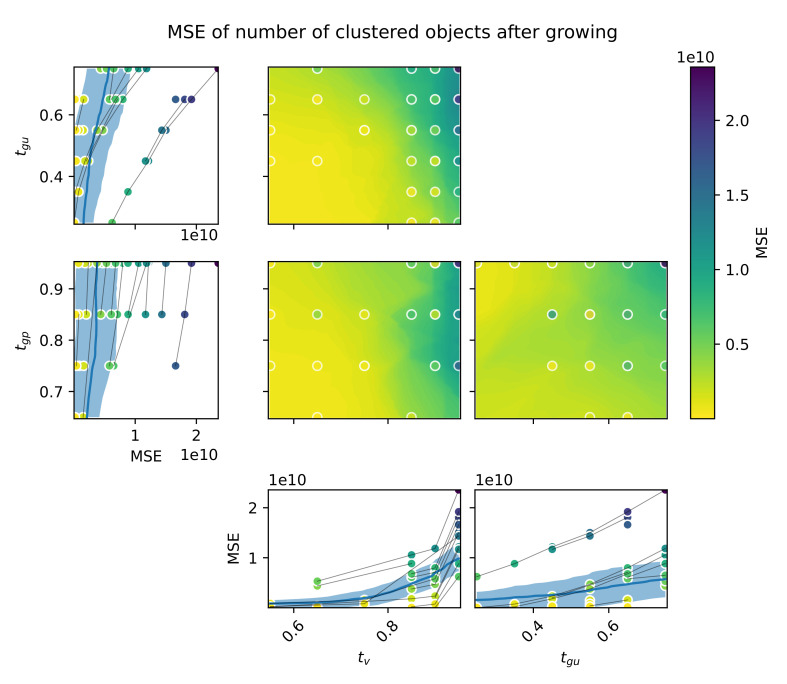
MSE of the number of grown objects as a function of the simulation hyperparameters after one step of the simulation. It mainly depends on the validation threshold $t_{v}$ and the unpure page threshold $t_{gu}$.

**Figure A3:**
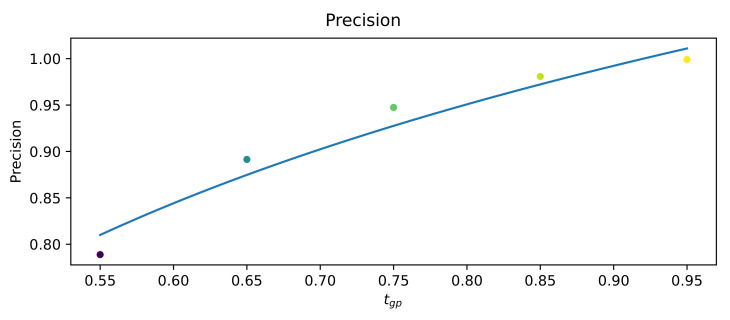
Dependence of annotation precision on $t_{gp}$.

**Figure A4:**
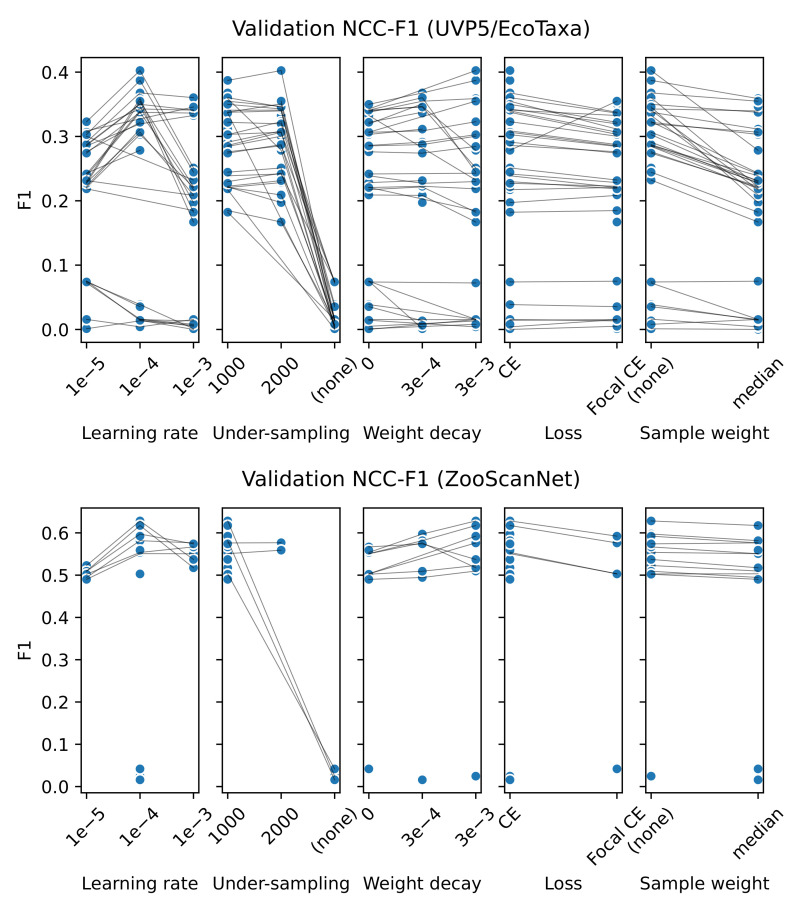
Dependence of the validation NCC-F1 for supervised learning on under-sampling, loss, weight decay, learning rate and sample weighting for the UVP5/EcoTaxa (**top**) and the ZooScanNet (**bottom**) datasets.

**Figure A5:**
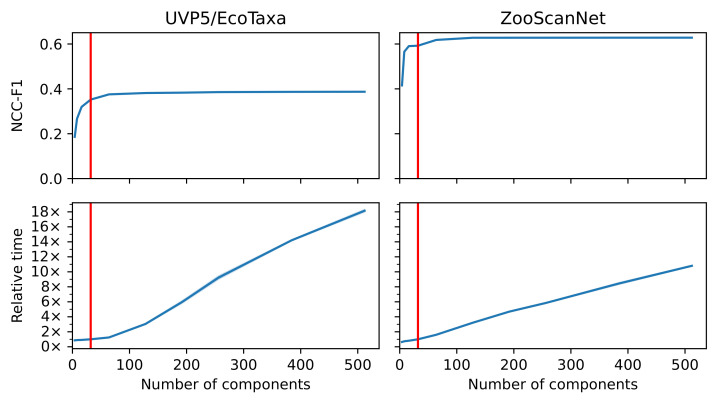
Time to predict and NCC-F1 in relation to different feature dimensionalities. Time is given in units relative to the time required to predict with 32 dimensions. The selected value of 32 dimensions is highlighted in red.

**Figure A6:**
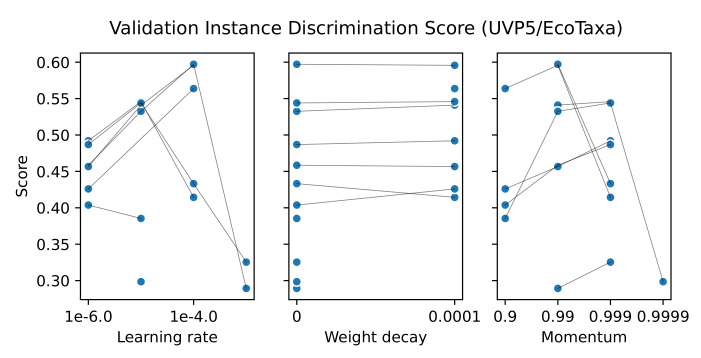

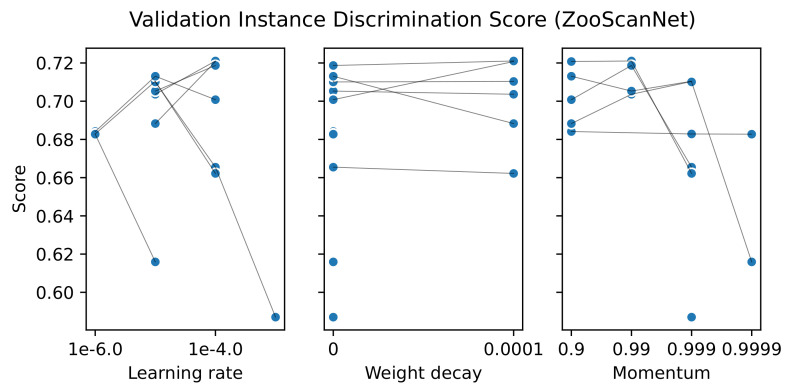
Dependence of the validation instance discrimination score on learning rate, weight decay and momentum for the UVP5/EcoTaxa (**top**) and the ZooScanNet (**bottom**) dataset.

**Figure A7:**
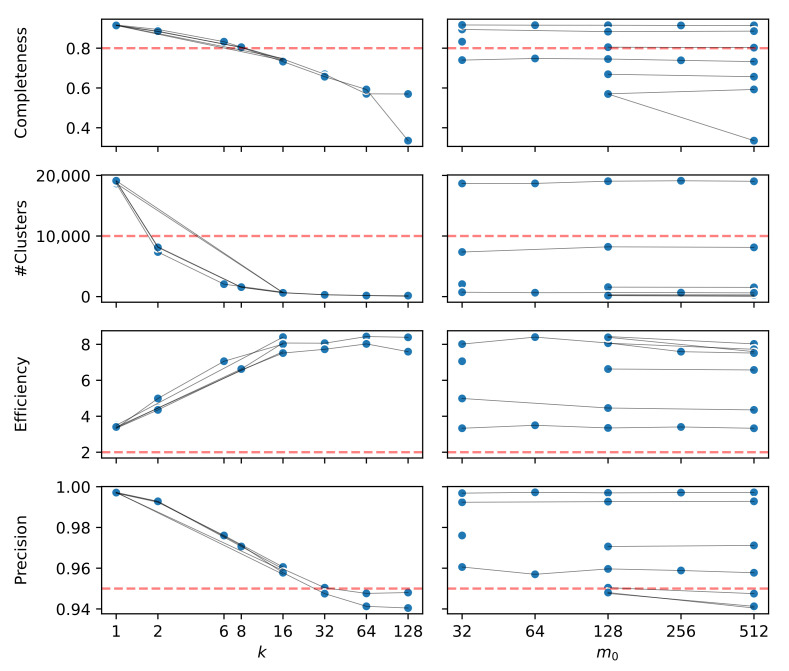
HDBSCAN*: Dependency of efficiency, completeness, number of resulting clusters and sorting precision on neighborhood size *k* and initial cluster size $m_{0}$. Each dot is an experiment with an individual hyperparameter configuration. In each plot, gray lines connect the dots that share the same hyperparameter configuration apart from the parameter that is varied in each column. *k* and $m_{0}$ are on a logarithmic scale.

**Figure A8:**
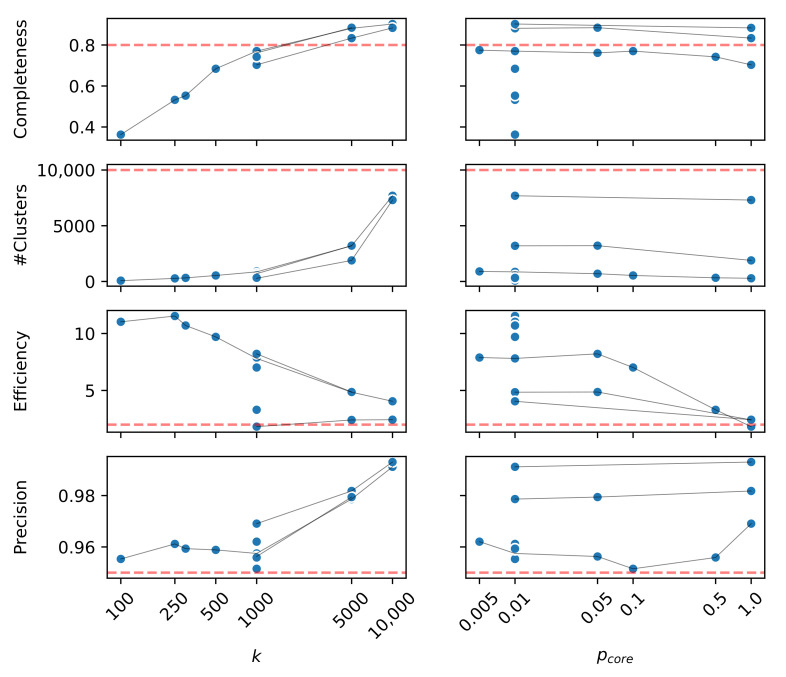
Shrunken *k*-means: Dependency of completeness, number of resulting clusters, efficiency and sorting precision on number of clusters *k* and core fraction $p_{\mathrm{core}}$. *k* and $p_{\mathrm{core}}$ are on a logarithmic scale.

**Figure A9:**
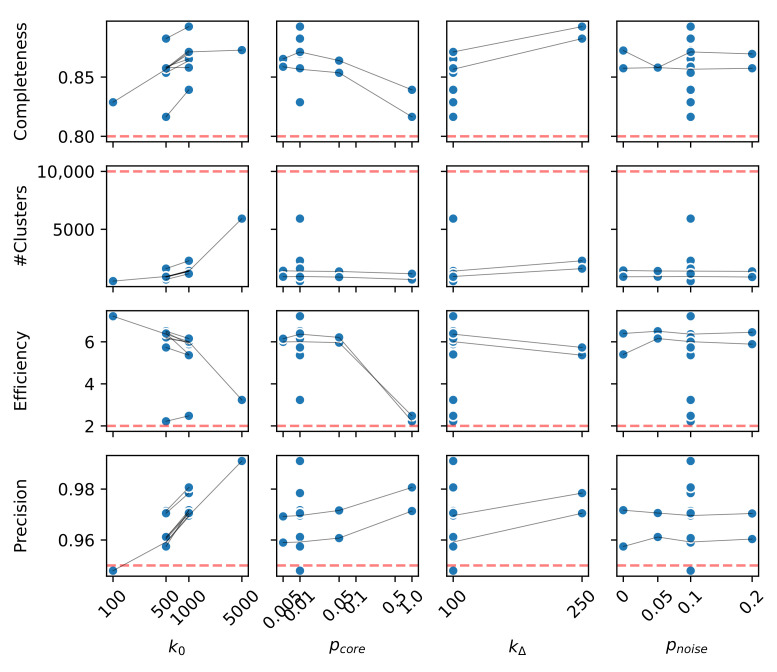
Partially labeled *k*-means: Dependency of completeness, number of resulting clusters, efficiency and sorting precision on noise fraction $p_{\mathrm{noise}}$, cluster count increment $k_{\Delta }$, initial number of clusters $k_{0}$ and and core fraction $p_{\mathrm{core}}$.

**Figure A10:**
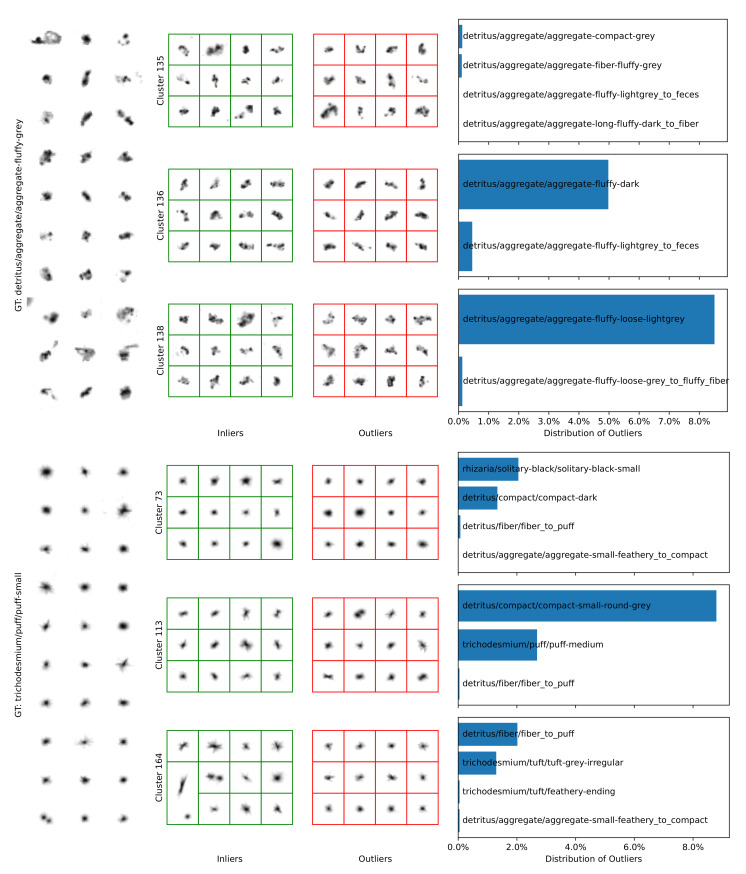
Inliers and outliers for two gold standard classes of the UVP5/MorphoCluster dataset after being assigned to clusters. The outliers are visually very similar to the inliers in the respective cluster and the outlier labels are plausibly close to the respective gold standard label.

## Data Availability

The data presented in this study are openly available at https://doi.org/10.17882/73002 (UVP5), https://doi.org/10.17882/55741 (ZooScanNet), http://dx.doi.org/10.1575/1912/7341 (WHOI-Plankton) and https://doi.org/10.7289/v5d21vjd (Kaggle).

## Abbreviations

    The following abbreviations are used in this manuscript:

MC20	The first implementation and evaluation of MorphoCluster.
NCC-F1	F1 score of a nearest centroid classifier.
UVP5/ET	UVP5/EcoTaxa, a coarsely annotated subset of the UVP5 dataset.
UVP5/MC	UVP5/MorphoCluster, a fine-grained annotation of the UVP5 dataset.

## Appendix A. Simulation of the MorphoCluster Process

The growing algorithm is outlined in Algorithms A1 and A2.

The required number of virtual clicks is recorded, according to Table A1.

**Table A1:** Actions that are counted as one click in the simulation.

Validation of one cluster core (accept/decline)
Removal of one outlier in cluster core
Validation of one page of recommendations (accept/decline)
Removal of one outlier in page of recommendations
Naming of one cluster
Naming of one remaining object

**Algorithm A1:** Growing: Initialization and Exponential Search

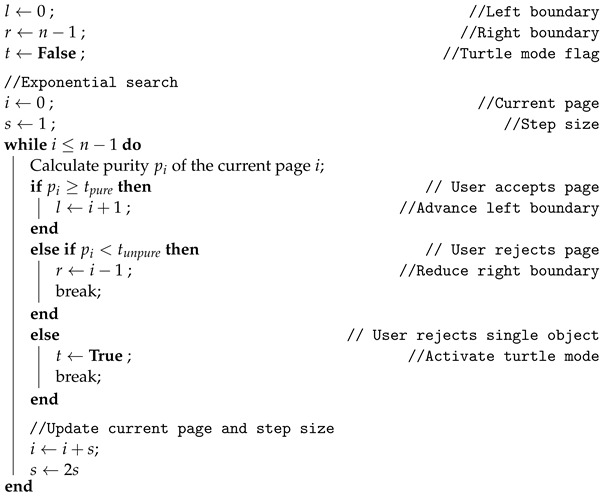

**Algorithm A2:** Growing: Binary Search and Turtle Mode

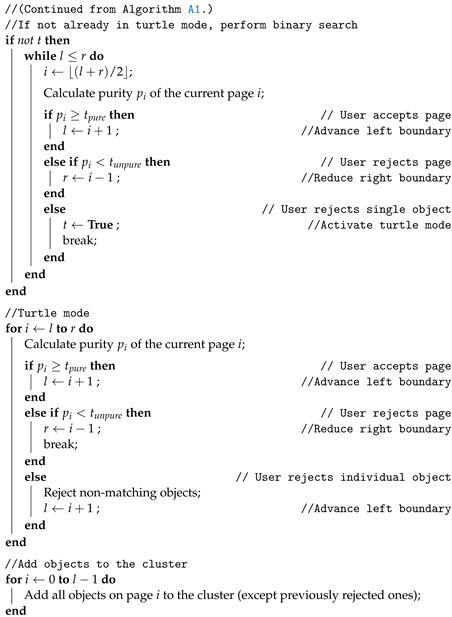

The behavior of a simulated used is approximated using validation threshold $t_{v}$, pure batch threshold $t_{gp}$ and unpure batch threshold $t_{gu}$ which influence the number of objects sorted in each step. A high validation threshold $t_{v}$ leads to fewer validated clusters and therefore a smaller number of objects added in both steps (and, by extension, a larger number of objects left to sort in further iterations). A high pure batch threshold $t_{gp}$ leads to fewer objects being sorted in the efficient exponential and binary search phase of the growing step but results in fewer false positives. The smaller the unpure batch threshold $t_{gu}$, the more objects get sorted in the less efficient linear search phase (“turtle mode”). The fewer objects are validated in one step, the more objects are left for the following steps. However, due to the decreasing cluster size setting, these following steps are less and less suitable to sort large numbers of objects efficiently.

The goal of the following experiments is to match the number of sorted objects in each step of the simulation as closely as possible to the progress and precision of the manual annotation process to find a single realistic validation and growing configuration that allows us to compare different changes to the system in later experiments.

We investigate only a few discrete parameter values because a continuous optimization of the simulation parameters would require a prohibitively large number of simulation runs. Furthermore, because multiple contradictory objectives (number of validated objects, number of grown objects, precision) need to be optimized at the same time, no single optimal solution exists and we manually pick a configuration that satisfies all three objectives adequately.

### Appendix A.1. Choosing t_v_ and t_gu_

First, we will optimize the validation threshold $t_{v}$ and unpure page threshold $t_{gu}$ to approximate the number of objects after validation and growing. The effect of both parameters can be observed directly in the first step of the annotation process. Furthermore, we need to limit the runtime of the individual experiments to obtain a high number of data points. Therefore, we restrict this optimization only to this first step.

The smallest discrepancy for the number of sorted objects after validation in the first step can be achieved with $t_{v}\geq 0.85$ (Figure A1). The smallest discrepancy for the number of sorted objects after growing is achieved with $t_{v}\leq 0.90$ and small $t_{gu}\leq 0.55$ (Figure A2).

For the following experiments, we therefore select $t_{v}=0.85$ and $t_{gu}=0.55$.

### Appendix A.2. Choosing t_gp_

The last parameter to determine is the pure page threshold $t_{gp}$ which influences the sorting efficiency and precision. In MC20, we measured the precision by evaluating gold-standard labeled objects hidden in the dataset [22]. Here, we try to match this value by grouping clusters of the same gold standard class. Figure A3 shows the dependence of annotation precision on $t_{gp}$. To approximate the sorting precision of 95% of the manual run [22], we set $t_{gp}=0.75$. Furthermore, this configuration also results in a balanced use of fast exponential and binary search (“rabbit mode”) and slow linear search (“turtle mode”).

## Appendix B. Representation Learning for MorphoCluster

After multiple feature extractor models have been trained with different hyperparameters, a “winning” configuration has to be selected for the the subsequent steps that optimizes the respective target score (NCC-F1 for supervised training, instance discrimination score for unsupervised training). For that, we use internal validation, i.e., we use the validation split of the source dataset to measure the target score. Only after selecting a specific feature extractor model do we apply it to the target dataset.

### Appendix B.1. Supervised Training of the UVP5 and ZooScanNet Models

We investigated how the learning rate, random under-sampling, reweighting of samples, training loss and weight decay impact the performance of a supervised feature extractor.

We performed more than 30 individual trials for each dataset with different hyperparameter configurations for the learning rate, random undersampling, sample reweighting, training loss and weight decay drawn randomly from a grid while observing the validation NCC-F1 as a measure of the quality of the image representations.

The learning rate determines the step size of the gradient descent optimizer.

Random under-sampling of the majority classes is used to balance the sizes of the different classes in order to train an unbiased classifier. Every epoch, a new class-stratified sample is drawn from the training set so that it contains at most $n_{c}^{*}$ examples from each class, respectively. One side-effect is a reduced training time because a smaller number of examples are processed in each epoch.

The reweighting of samples is a different approach to combat class imbalance in order to obtain an unbiased classifier [83]. Instead of under-sampling majority classes, their data points are assigned lower weights to reduce their influence. Here, we compute the weight $w_{c}$ of a class *c* inversely proportionally to its size $n_{c}$:

(A1) $$w_{c}=\frac{{\mathrm{median}}_{d\in C}n_{d}}{n_{c}}$$

Accordingly, the influence of individual samples is increased ($w_{c}>1$) in classes smaller than the median class size, and decreased ($w_{c}<1$) in classes larger than the median.

As the training loss, we investigate focal loss [84] in addition to the commonly used cross-entropy loss (CE). It is an adaptation of CE to down-weight the influence of already well-classified examples.

Weight decay [85] is a regularization technique to reduce the complexity of the network in order to improve the generalization of the model [86].

#### Results

The median runtime of a training run was 29.41 h for ZooScanNet and 9.43 h for UVP5/EcoTaxa.

Figure A4 shows the dependence of the validation NCC-F1 on under-sampling, loss, weight decay, learning rate and sample weighting for the UVP5/EcoTaxa and ZooScanNet datasets.

Throughout all trials, the most robust settings, as assessed using the NCC-F1, are a learning rate of 1 × 10^−4^, a weight decay of 3 × 10^−3^, cross-entropy loss, undersampling to up to 1000 objects per class and no sample reweighting.The models with these hyperparameters are selected for the experiments.

### Appendix B.2. Dimensionality Reduction

After having selected suitable hyperparameters for the training of a supervised model, we optimized the three dimensionality reduction approaches. We investigated the relationship between the dimensionality of features and the runtime and F1 score of the corresponding nearest centroid classifier with PCA dimensionality reduction. We also optimized the learning rate of the two trained dimensionality reduction approaches. For evaluation, we used the validation split of the respective dataset that the feature extractor was trained on.

We trained the single-stage and multi-stage models for a reduction to 32 features, varying the learning rate. The other hyperparameters were set to the previously selected values. The second stage in the multi-stage condition was trained for only 250 epochs.

#### Results

As apparent in Figure A5, the time to predict grows proportionally with the feature dimensionality. The NCC-F1 saturates at around 32 dimensions for both datasets. While a further reduction to 16, 8 or 4 dimensions decreases the runtime by up to 24%, it also considerably impairs the quality of the image representations by up to 17 percentage points. A further increase to 64 dimensions only provides a small gain of 2.50 percentage points while taking 21% longer. For ZooScanNet, even a reduction to 16 dimensions seems to be feasible without impairing the feature quality too much (impairment by 0.20 percentage points). We conclude that the initial choice of d=32 is justified.

The single-stage model performs best when trained with a learning rate of 1 × 10^−4^ which matches the learning rate selected for the full-dimensionality training. In accordance with the common practice of fine-tuning at a lower learning rate, the training of the second stage of the multi-stage condition exhibits the best performance, with a learning rate of 1 × 10^−5^.

Training was often unstable in the single-stage condition. As a result of the additional training stage, the multi-stage approach took nearly twice as long as one full training.

### Appendix B.3. Unsupervised Training of the UVP5 and ZooScanNet Models

For unsupervised representation learning, we used the momentum contrast (MoCo) approach by He et al. [37], with the refinements (more data augmentation, an additional MLP layer and a cosine learning rate schedule) of Chen et al.(MoCo v2) [38]). In addition to the *learning rate* and *weight decay* (Appendix B.1), we varied the momentum coefficient *m*. We set the temperature parameter τ to 0.07, like in [37].

#### Appendix B.3.1. Unsupervised Model Selection

As well as in training, the availability of labeled target domain data also plays a role in model selection. “The point of unsupervised learning […] is that there is no access to the labels, as, otherwise, we could incorporate them and would have to compare to semi-supervised and fully supervised methods.” [87]. This obviously also extends to the model selection step to avoid biasing the results of a study by tuning hyperparameters based on supervised metrics [87]. (However, this subtlety is regularly disregarded to simplify the model evaluation; see e.g., [37,39].) Moreover, problem-agnostic unsupervised scores are not likely to work well in practice [87].

For unsupervised model selection, we therefore defined the task-specific instance discrimination score that does not require labeled data. It is specific to the instance discrimination task that we use for unsupervised training, but independent from training hyperparameters. Similar to the utilized MoCo training loss, it is based on two different views of the same object. We compare the representation of the raw, unaltered image to the representation of an image that was rotated and/or flipped randomly.

For an individual object *i*, we define the per-object instance discrimination score as

(A2) $$s\left(i\right)=\frac{b(i)-a(i)}{\max\{a(i),b(i)\}}$$

where a(i) is the distance between the raw and the perturbed representation of an object *i* and b(i) is the distance of object *i* to its nearest neighbor. (This formulation is similar to the silhouette score [88] but serves a different purpose.) The instance discrimination score is then the average of the s(i) for all objects in the whole dataset. The instance discrimination score ranges from −1 to 1 and favors representation spaces where both views of an object are more similar than an object to its nearest neighbor, i.e., representation spaces that are discriminative of individual objects while being transformation-invariant.

These properties will facilitate the later unsupervised partition of the representation space into visually distinct and homogeneous clusters, just like its supervised equivalent NCC-F1 (Section 2.4).

For practical reasons, we use an approximation of the nearest neighbor distance b(i), where each object is compared only to a small sample of the whole dataset. (In our implementation, we process the query objects in batches of 1000 and draw 50 k new neighbor candidates for each batch. We did not observe any significant fluctuations despite the stochasticity of the approach.)

The instance discrimination score is used for unsupervised model selection.

#### Appendix B.3.2. Results

We performed more than 15 individual trials for each dataset with different hyperparameter configurations drawn randomly from a grid while observing the validation instance discrimination score as a measure of the quality of the image representations. The median runtime of a trial was 29.67 h for ZooScanNet and 18.86 h for UVP5/EcoTaxa. Fewer trials were conducted because training a MoCo model on UVP5/EcoTaxa takes significantly longer than training a simpler classifier, for which, the objects of large classes can be undersampled.

Figure A6 shows the dependence of the NCC-F1 on the learning rate, weight decay and momentum for the UVP5/EcoTaxa (top) and the ZooScanNet (bottom) dataset. For both UVP5/EcoTaxa and ZooScanNet, the best results are achieved with a momentum of 0.99, a weight decay of 0.0001 and a learning rate of 1 × 10^−4^.

Our observations regarding momentum and weight decay match those of He et al. [37]. This means that these hyperparameters are insensitive to the dataset. Our implementation differs in the choice of the optimizer (Adam vs. SDG), which is why a different learning rate is optimal.

## Appendix C. Shrunken *k*-Means (S-*k*-Means)

The algorithm for S-*k*-means is given in Algorithm A3.

**Algorithm A3:** Shrunken *k*-means

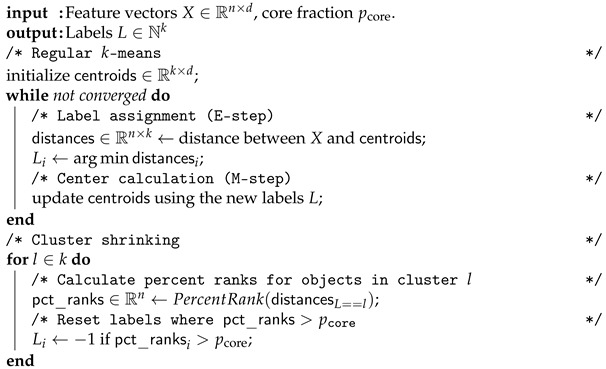

## Appendix D. Partially Labeled *k*-Means (PL-*k*-Means)

The algorithm for PL-*k*-means is given in Algorithm A4.

**Algorithm A4:** Partially labeled *k*-means

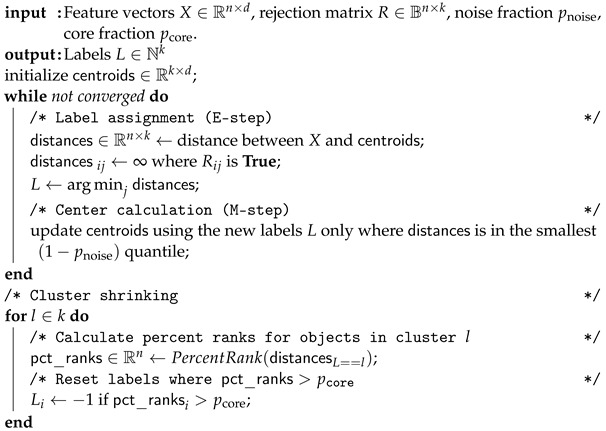

## Appendix E. Optimization of Clustering Parameters

To select viable hyperparameters for each clustering algorithm, we run the simulation with different parameter choices using the UVP5/MC dataset and the original features from MC20.

### Appendix E.1. Optimization of HDBSCAN*

We varied the initial cluster size $m_{0}$ and the neighborhood size *k*. As seen in Figure A7, the target metrics are mainly influenced by the neighborhood size *k*, whereas changes in the initial cluster size $m_{0}$ do not have a substantial effect. The completeness decreases steadily with a growing *k*. While very high for small k≤2, the total number of clusters quickly approaches a lower bound of 500 for larger *k*. The efficiency increases with *k* to an upper bound of 8 at k=16. The precision steadily decreases with greater *k* and falls below 95% at k=32.

The size of the calculated clusters obviously depends on the size of the dataset. However, the experiment shows that the overall outcome is largely independent of the actual choice of the initial cluster size $m_{0}$. We, therefore, conclude that a single $m_{0}$ should work with a wide range of dataset sizes.

Deviating from MC20 [22], we select k=8 and $m_{0}=128$.

### Appendix E.2. Optimization of Shrunken k-Means

We varied the number of clusters *k* and the core fraction $p_{\mathrm{core}}$. Figure A8 shows the results of this experiment. The outcome is primarily influenced by the cluster size *k*. Smaller k<1000 lead to a small number of clusters and a high efficiency, but also to a low completeness and lower precision. The completeness, total number of clusters and precision grow with *k*. The completeness surpasses 0.5 at k=250. The total number of clusters remains small and starts to grow more substantially after k=1000. The efficiency is greatest at k≤250 and $p_{\mathrm{core}}\leq 0.05$ and decreases with a growing *k* and $p_{\mathrm{core}}$. The precision is above 95% for all configurations and approaches 99% for very large k≥5000.

The effect of the value of $p_{\mathrm{core}}$ on the efficiency shows that cluster shrinking is in fact beneficial to removing outliers from the proposed clusters.

We select k=1000 and $p_{\mathrm{core}}=0.01$.

### Appendix E.3. Optimization of Partially Labeled k-Means

We varied the noise fraction $p_{\mathrm{noise}}$, the cluster count increment $k_{\Delta }$, the initial number of clusters $k_{0}$ and the core fraction $p_{\mathrm{core}}$. Figure A9 shows the results of this experiment.

A smaller cluster count increment of $k_{\Delta }=100$ leads to a slightly better sorting efficiency and a lower number of clusters, but also to a smaller completeness. A small core fraction $p_{\mathrm{core}}$ leads to a larger number of validated objects and a higher sorting efficiency, but also a slightly smaller sorting precision, whereas the number of clusters is virtually unaffected.

The most influential parameter is $k_{0}$, followed by $p_{\mathrm{core}}$ and $k_{\Delta }$, whereas $p_{\mathrm{noise}}$ seems to have no influence on the outcome. Smaller $k_{0}$ leads to fewer clusters and a higher sorting efficiency, but also fewer validated objects and a lower precision. The completeness is above 80% for all configurations. It grows with $k_{0}$ and $k_{\Delta }$ and falls with $p_{\mathrm{core}}$. The total number of clusters grows with $k_{0}$ and, to a lesser extent, with $k_{\Delta }$, and decreases slightly with $p_{\mathrm{core}}$. The efficiency decreases with a growing $k_{0}$, $p_{\mathrm{core}}$ and, to a lesser extent, with $k_{\Delta }$ from above 6 to 2. Like for S-*k*-means and HDBSCAN*, the precision is above 95% for all configurations. It grows with $k_{0}$, $p_{\mathrm{core}}$ and $k_{\Delta }$.

Like for S-*k*-means, the effect of the value of $p_{\mathrm{core}}$ on the efficiency shows that cluster shrinking is beneficial to removing outliers from the proposed clusters. The outlier removal during the re-calculation of the cluster centers controlled by $p_{\mathrm{noise}}$ seems to play a minor role.

We select $p_{\mathrm{noise}}=0.1$, $k_{\Delta }=100$, $k_{0}=500$ and $p_{\mathrm{core}}=0.01$.

## Appendix F. Visual Similarity and Conceptual Closeness of Cluster Outliers and Gold Standard

Figure A10 shows the respective inliers and outliers in three clusters for two gold standard classes of the UVP5/MorphoCluster dataset after being processed in the simulation using HDBSCAN* and supervised image representations.

The visual similarity and the conceptual closeness of the outlier labels to the gold standard labels underlines the subjectivity and fuzzyness of the annotations.
